# Tumor associated macrophages-derived exosomes facilitate hepatocellular carcinoma malignance by transferring lncMMPA to tumor cells and activating glycolysis pathway

**DOI:** 10.1186/s13046-022-02458-3

**Published:** 2022-08-19

**Authors:** Minghao Xu, Chenhao Zhou, Jialei Weng, Zhaoshuo Chen, Qiang Zhou, Jian Gao, Guoming Shi, Aiwu Ke, Ning Ren, Huichuan Sun, Yinghao Shen

**Affiliations:** 1 Department of Liver Surgery and Transplantation, Liver Cancer Institute and Zhongshan Hospital, Fudan University, No.180, Fenglin Road, Shanghai, 200032 China; 2 Department of Hepatobiliary Pancreatic Surgery, Fujian Medical University Cancer Hospital, Fuzhou, 350014 China; 3 Department of Thoracic Surgery, Zhongshan Hospital, Fudan University, Shanghai, 200032 China

**Keywords:** LncMMPA, Hepatocellular carcinoma, Tumor associated macrophage, Metabolic reprogramming

## Abstract

**Background:**

Tumor-associated macrophages (TAMs), which form a large part of the tumor microenvironment, are normally regulated by metabolic reprogramming. However, the potential mechanisms of the immune-metabolism interaction between hepatocellular carcinoma (HCC) cells and TAMs remain unclear.

**Methods:**

The candidate long non-coding RNAs (lncRNAs) were screened by Smart-seq based scRNA-seq method and then validated by qPCR. Immunostaining analysis was done to examine the levels of markers for TAMs and glycolysis. Exosomes from primary TAMs of human HCC tissues were isolated by centrifugation, and their internalization with lncRNAs was confirmed by immunofluorescence. The underlying mechanism of TAMs-derived exosomal lncRNA to HCC was confirmed by luciferase reporter assay and RNA immunoprecipitation. Metabolism regulation was evaluated through glucose consumption, lactate productions and extracellular acidification rates (ECARs). Mouse xenograft models were used to elucidate the in vivo effect of candidate lncRNAs on tumor growth.

**Results:**

TAMs augment the aerobic glycolysis in HCC cells and their proliferation by the extracellular exosome transmission of a myeloid-derived lncRNA, M2 macrophage polarization associated lncRNA (lncMMPA). Mechanistically, lncMMPA not only could polarize M2 macrophage, but also could act as an microRNA sponge to interact with miR-548 s and increase the mRNA level of ALDH1A3, then further promote glucose metabolism and cell proliferation in HCC. Moreover, lncMMPA increased HCC cell multiplication through interacting with miR-548 s in vivo. Clinically, lncMMPA expression associates with glycolysis in TAMs and reduced survival of HCC patients.

**Conclusion:**

LncMMPA plays an important role in regulating HCC malignancy and metabolic reprogramming of miR-548 s/ALDH1A3 pathway.

**Supplementary Information:**

The online version contains supplementary material available at 10.1186/s13046-022-02458-3.

## Background

Hepatocellular carcinoma (HCC), the most common primary liver cancer, is the third leading cause of cancer-related mortality worldwide [1]. Although treatments with chemotherapy, immunotherapy and targeted therapy are benefiting more and more patients with HCC, overall survival of these patients remains dismal [2–4]. In this context, the dysregulation of immune regulation and metabolic reprogramming plays a vital role both in the tumor microenvironment (TME) and at the host level [5, 6], and closely associated with the progression and treatment efficacy in most solid tumors [7]. Therefore, it is important to reveal the relationship between tumor immune-metabolism microenvironment and tumor cells and develop more effective strategies to treat HCC. Tumor suppressive macrophage (M1 type) and tumor-supportive macrophages (M2 type) constituted the major cell types of tumor microenvironment, and M2 macrophage-associated markers mostly associate with a poor clinical outcome in different types of solid tumors [8]. It is well known that M2 macrophages act as a driving factor in TAMs, which usually promote tumor growth, malignance, and metastasis [9]. Tumor-derived exosomes can travel to the tumor microenvironment and promote the polarization of M2 macrophage, then eventually accelerate tumor progression and metastasis [10, 11]. Exosomes released from macrophages in ovarian cancer have also been studied to trigger immune imbalance in the tumor microenvironment [12]. However, no information is available on bioactive transmission by TAMs-derived exosomes to tumor cells in HCC.

Exosomes generally contains nucleic acids (miRNA, mRNA, other noncoding RNA and DNA), proteins and lipids [13], thereby mediating communication in the tumor microenvironment to HCC development and progression [14]. Long non-coding RNAs (lncRNAs) are non-coding RNAs longer than 200 nucleotides. Numerous researches have shown that epigenetic modification, including long noncoding RNAs (lncRNAs), may play important roles in hepatocellular carcinoma (HCC) initiation and progression as well as in remodeling microenvironment of HCC. LncRNA TUC339, which is from HCC exosomes, has been reported to mediate macrophage activation and polarization [15]. In addition, the antisense of lncRNA distal-less homeobox 6 (DLX6-AS1), not only acts as a prognostic biomarker in liver cancer [16], but also the stemness of cancer cells in HCC patients [17].

Metabolism reprogramming is a common feature of HCC [18], dominated by elevated aerobic glycolysis of HCC cells, referred to as the Warburg effect [19]. Aerobic glycolysis efficiently generates energy and macromolecules required for cell growth, thus contributing to the rapid growth and proliferation in tumors [20]. Thus, much attention has been paid to glycolytic regulation during tumorigenesis, in particularly, glycolysis-related genes [21]. However, little is known about the contributions of lncRNAs to HCC metabolism reprogramming and malignancy.

In this study, we found that TAMs-derived exosomes play a role in the regulation of glucose metabolism and cell proliferation in HCC cells. In addition, we examined the role played by the exosome-packaged lncMMPA, originating from TAMs, in controlling the aerobic glycolysis and cell proliferation of hepatocellular carcinoma tumor cells and studied the underlying mechanisms.

## Material methods

### Patient specimens and TAMs isolation

We have studied three cohorts of patients with HCC who had surgery at Zhongshan Hospital (ZSHS cohort). These cohorts comprise randomly selected cohort 1 with 108 formalin-fixed paraffin-embedded tissues, cohort 2, and cohort 3 with 40 and 117 fresh tissues, respectively. The ethics committee of Zhongshan Hospital has given its approval for this study’s protocol. All the participants in this research work gave their informed consent in writing. The research work described here was conducted following the provisions of the Declaration of Helsinki of 1975.

TAMs were separated from fresh HCC tumor samples. Small pieces (1–2 mm) of the tissues were made followed by 2 h digestion with 5% fetal bovine serum DMEM with collagenase VIII-DNase I (Sigma-Aldrich) at 37 °C. The released cells were filtered through cell strainers with a pore size of 70 μm. The cell suspension (1 ml) was layered on top of a Percoll (GE Healthcare) gradient, consisting of 40% Percoll (5 ml) and 60% Percoll (5 ml) at the bottom in a 15-ml tube. The tubes were then centrifuged at 2500 rpm for 20 min. The cell layer at in-between 40 and 60% Percoll layers contained TAMs.

### Cell culture conditions and treatment

Hep3B and BEL7404 cell-lines (American Type Culture Collection) and were grown employing standard methods. Cell culture media were as recommended by the supplier, with 10% fetal bovine serum (FBS) supplementation and incubations were in chambers maintained in an atmosphere of 5% CO_2_ and 95% air, and at 37 °C.

Peripheral blood monocyte cells obtained from people with normal health were separated by Ficoll density gradient centrifugation at 450 g at 20 °C for 20 min. The 15-ml centrifuge tubes contained 5 ml of Ficoll (GE Healthcare) at the bottom and 10 ml of blood cell suspension on the top. Monocytes, which migrated in-between the layers of plasma and Ficoll, were collected. Monocytes were incubated with 100 ng/ml of PMA for 48 h to obtain human monocyte-derived macrophages (MDMs). DMEM culture medium plus 10% fetal bovine serum supplemented with and penicillin and streptomycin were used to grow MDMs. The MDMs were treated with 100 ng/ml of LPS for the induction of M1 macrophages and 20 ng/ml of IL-4 for the induction of M2 macrophages.

HCC cancer cells (3 × 10^5^ cells) were transferred to the lower chamber of a 12-well Transwell system, while the upper chamber was added with macrophages (3 × 10^5^ cells). The pore size of upper chamber was 0.4 μm. Following co-culture for 6 days, the macrophages were removed and after washing once with phosphate buffered saline, the HCC cells were cultured in freshly added growth medium.

### Hematoxylin and eosin (H&E) and immunohistochemistry (IHC) assay

Tissue samples from ZSHS cohort 1 (*n* = 108) were embedded in blocks of paraffin, followed by preparation of 4-mm thick sections using a microtome and stained with H&E (Sigma-Aldrich, St. Louis, MO, USA) for histopathological investigation. For IHC staining, rehydration of paraffin-embedded tissue sections was done in sodium citrate buffer (pH 6.0), followed by heat-mediated antigen retrieval employing a microwave. Tissue sections were then transferred in to blocking buffer for 2 h followed by incubation with antibodies against CD68 (Abcam; ab955), CD163 (Abcam; ab182422), HK2 (Abcam; ab209847), GLUT1 (Abcam; ab115730) in a humidified box at 4 °C for 24 h. Tissue sections were then washed and incubations with secondary antibody (Proteintech Group, Inc., Wuhan, China; PK10006) were done. Immunodetection was performed using DAB. The IHC score is based on staining intensity on a scale of 1 to 4 (absent = 1, weak = 2, moderate = 3, or strong = 4). An average intensity score per tumor from 3 independent fields was calculated for each patient.

### Transient transfection and transduction

Transfections were conducted with the Lipofectamine 3000 (Invitrogen) as per the supplier’s protocol. The microRNA mimics, microRNA inhibitors and their corresponding negative control RNAs (GenePharma) were transfected into the cells at 50 nM final concentration.

Transduction of macrophages (3 × 10^5^ cells per well) using lentiviral particles (MOI of 100) with 5 μg/ml Polybrene was conducted in 24-well plates. The nucleotide sequences of microRNA mimics, microRNA inhibitors, shRNAs and their corresponding negative control are given in Supplementary Table S1.

### Determination of glucose consumption, lactate production and ECAR

HCC cells were seeded into 6-well plates with freshly added growth medium and incubated for 24 h. Then the incubation media were collected, and kits from Biovision were employed to determine consumption of glucose and production of lactate, as detailed in the manufacturer’s brochure. Cell number was calculated with a cell counter. The seahorse extracellular Flux analyzer XF96 (Seahorse Bioscience) was employed to determine metabolic changes in cells, in vitro, as mentioned in the manufacturer’s instructions. Briefly, HCC cells with the treatments, as specified, were transferred into 96-well culture plates followed by incubation at 37 °C overnight. Then the cells were used for measurement of ECAR. After measurement of baseline concentration, glucose, oligomycin, and 2-deoxyglucose were added sequentially into each well at the indicated time points for ECAR measurement. The measured ECAR values were adjusted to the total protein content and shown as mpH/min. ECAR measurements are shown as the mean ± s.d. of experimental triplicates.

### Cell proliferation analyses and apoptosis assay

Determination of cell proliferation was done with cell-counting kit-8 (Dojindo) as per the instructions of manufacturer. In 96-well plates, cells were first seeded and 10 μl of CCK8 solution was added at each time point, followed by 2 h incubation in dark. After the incubations, absorbance was measured at 450 nm.

As for apoptosis assay, cells were incubated with Adriamycin (2 μg/ml) for 48 h, and then treated with trypsin-EDTA to dissociate, followed by centrifugation, to collect the cells. Annexin V Apoptosis Detection kit (eBioscience) and flow cytometry (BD Biosciences) was used for measuring apoptosis.

### Exosome experiment

Exosomes were separated from the cell culture medium by ultracentrifugation. First, the medium was centrifuged at 3000 rpm for 10 min at 4 °C. The supernatant was centrifuged at 10,000 g for 30 min at 4 °C and the cellular debris pellet was removed. Then the resulting supernatant was passed through a 0.22 μm filter, followed by ultracentrifugation at 100,000 g at 4 °C for 90 min, and the resulting pellet contained exosomes.

Exosomes were first quantified by NanoSight NS300 instrument (Malvern Instruments) and electron microscopy was used to assess their purity. Primary antibodies against Alix, CD9 and TSG101 were used for the identification of exosomes.

Equal numbers of exosomes derived from MDMs or TAMs were treated with RNaseA and TritonX-100 treatment, and then RNA was extracted with TRIzol and normalized to cel-miR-39-3p for qRT-PCR. In vitro studies were done by treating HCC cells with 50 μg/mL exosomes.

For the visualization of lncRNA and exosome co-localization, FAM-labeled lncMMPA (in vitro transcription) was introduced into exosomes by electroporation using a GenePulser Xcell electroporation system (Bio-Rad), followed by the labeling of exosomes with Dil (Beyotime Biotechnology). Hep3B cells were incubated with exosomes labeled with Dil followed by visualization using laser scanning confocal microscopy (TCS SP8, Leica).

### RNA extraction, qRT-PCR, and Western blotting

Extraction of total RNA from indicated tumor tissues or cell lines was done using TRIzol reagent (Takara). Employing 1 μg of total RNA and PrimeScript RT Reagent Kit (Takara), the first-strand cDNA was synthesized. The levels of miRNA were determined by qRT-PCR with SYBR-Green ΙΙ PCR kit (Takara) and U6 snRNA was used as the reference. LncRNA and mRNA concentrations were measured employing SYBR-Green ΙΙ PCR kit (Takara). β-actin was used as the internal control. Supplementary Table S2 shows the sequences of the primers used.

Extraction of protein was from the HCC cells was done using RIPA buffer, and determined using the BCA Protein Assay Kit (Beyotime Biotechnology). Separation of extracted proteins (40 μg per lane) was done by SDS-polyacrylamide gel electrophoresis and the separated proteins were transferred to polyvinylidenedifluoride (PVDF) membranes (Bio-Rad). The PVDF membranes were blocked using 5% non-fat milk for 1 h and primary antibodies against GLUT1 (Biorbyt, St Louis, MO, USA; orb157188), HK2 (Abcam; ab227198), LDHA (Abcam; ab52488), ALDH1A3 (Proteintech; 29,373–1-AP), β-actin (Abcam; ab8226), Alix (Abcam; ab88743), CD9 (Abcam; ab236630) and TSG101 (Abcam; ab125011) were used. Secondary antibodies were labeled with Peroxidase. The ECL detection system (Thermo) was used for visualization.

### Luciferase reporter assay

Co-transfection of either pGLO, pGLO-lncMMPA, or pGLO-lncMMPA mut (miR-548 s binding site), or pGLO-ALDH1A3, or pGLO-ALDH1A3 mut (miR-548 s binding site) together with miR-548 s mimics or its negative control was done in Hep3B cells. Renilla luciferase activity was employed as the reference for normalizing the relative luciferase activity. A Dual-Luciferase Reporter Assay System (Promega) was used for determining the firefly luciferase and Renilla luciferase activities, according to the supplier’s instructions.

### RNA immunoprecipitation

HCC cells were co-transfected pSL-MS2, pSL-MS2-lncMMPA, or pSL-MS2- lncMMPA mut (miR-548 s binding site) with pMS2-FLAG. Cells were collected 48 h post-transfection, to conduct RNA immunoprecipitation (RIP) experiments. The RNA-protein complexes were immunoprecipitated using a 3xFLAG antibody (Sigma). The RNA fraction separated by RIP was analyzed by qRT-PCR.

### In vivo xenograft model

To elucidate the in vivo effect of lncMMPA on tumor growth, male BALB/c nude mice of 4-weeks age (Shanghai SLAC Laboratory Animal Co., Ltd.) were orthotopically injected with Hep3B cells into the right flank (*n* = 6 per group), subcutaneously, to create the HCC xenograft model. Seven days following inoculation, 30 μl of PBS suspension of exosomes (10 μg) prepared from MDMs with the indicated treatment was injected intratumorally every 3 days. For miR-548 s mimics treatment, 50 μl of OPTI-MEM containing miR-548 s mimics (100 pmol) and Lipofectamine 3000 (Thermofisher) mixture was injected intratumorally every 3 days. Tumor volume (mm^3^) was assessed using the following formula: Tumor volume (mm^3^) = longer diameter x shorter diameter^2^/2. After fixing in polyformaldehyde, xenografts were embedded in paraffin and processed for immunofluorescence staining for Ki67. The animal experiments were approved by the Zhongshan Hospital Animal Care and Use Committee.

### Immunofluorescence microscopy

For Ki-67 staining, paraffin-embedded samples were blocked with 1% bovine serum albumin in PBS for 30 min, and incubated with Ki-67 antibody (Abcam; ab243878). The secondary antibody used was Alexa Fluor® 488-conjugated goat anti-rabbit IgG. DAPI (C1002; Beyotime Biotechnology) was used to visualize cell nuclei. Slides were observed with a confocal microscopy (TCS SP8, Leica). Five images of each sample were used to count and calculate the percent of ki-67 positive cells.

### Statistical analysis

Mann-Whitney U test in GraphPad Prism was used to analyze the results. Data are shown as mean ± SEM. The single cell data was analyzed by the Python-based algorithm SCANPY (version 1.7.2). Uniform Manifold Approximation and Projection (UMAP) was run for visualization. Violin plots for marker genes were generated utilizing the stacked violin function as executed in SCANPY. To evaluate the differential genes between the conditions, we computed Cohen’s d statistic to estimate the magnitude of changes in gene expression within a cell population. Statistical analyses were conducted using R (v4.0.1). The type of statistical method employed for analysis is indicated in the text during the description of corresponding results and details for the test are elaborated in the legends of the relevant figures. Statistical significance was considered when *P*-value < 0.05.

## Results

### RP11-1100 L3.8 is identified as a modulator of macrophage M2 polarization in hepatocellular carcinoma

**Fig. 1:**
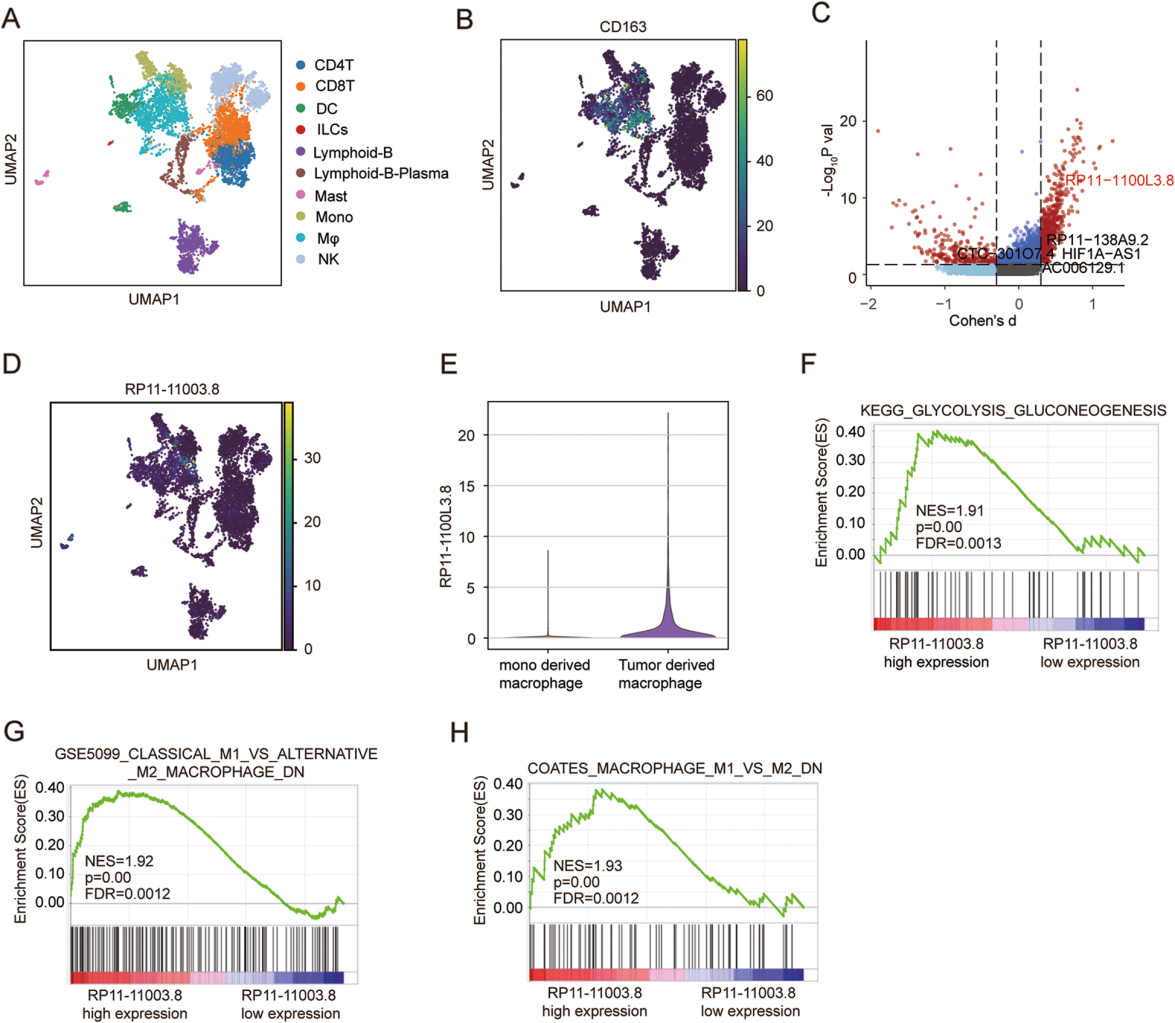
RP11-1100 L3.8 is identified as a modulator of macrophage M2 polarization in hepatocellular carcinoma. **A** UMAP plot of all cells colored by each cell type in HCC patients (GSE140228). **B** Expression of canonical marker genes for macrophages, as shown by UMAP plot. **C** Volcano plots show the differentially expressed genes between tumor cells and normal cells. The most significant expressed lncRNA RP11-1100 L3.8 was highlighted by red. **D** Expression of RP11-1100 L3.8 in each cell type of HCC, shown by UMAP plot. **E** Violin plot showing the expression of RP11-1100 L3.8 in tumor-derived macrophage and monocyte-derived macrophage. **F** GSEA data showing the enrichment of KEGG_GLYCOLYSIS_GLUCONEOGENESIS peaks in patients with high RP11-1100 L3.8 expression compared with patients with low RP11-1100 L3.8 expression in TCGA LIHC dataset. NES, normalized enrichment score; FDR, false discovery rate. **G** GSEA data showing the enrichment of geneset GSE5099_CLASSICAL_M1_VS_ALTERNATIVE_M2_MACROHPAGE_DN peaks in patients with high RP11-1100 L3.8 expression compared with patients with low RP11-1100 L3.8 expression in TCGA LIHC dataset. NES, normalized enrichment score; FDR, false discovery rate. **H** GSEA data showing the enrichment of COATES_MACROPHAGE_M1_VS_M2_DN peaks in patients with high RP11-1100 L3.8 expression compared with patients with low RP11-1100 L3.8 expression in TCGA LIHC dataset. NES, normalized enrichment score; FDR, false discovery rate

To examine the epigenetic correlation between infiltration of TAMs and advancement of HCC, Smart-seq based scRNA-seq method was applied to study tumor-derived CD45^+^ cells and four immune-relevant sites (adjacent liver, blood, ascites and hepatic LNs) of six treatment-naive liver cancer donors (data from GSE140228, Supplementary Fig. S1) [9]. Those cells were annotated by canonical marker genes as ten cell types include Dendritic cells (DC), CD4^+^T cells, Monocytes, CD8^+^T cells, Macrophages (Mφ), B cells, Natural killer cells, Plasma, Mast, and innate lymphoid cells (ILCs) as suggested by original study [9] (Fig. 1A). To investigate the lncRNA specifically expressed in the macrophages of HCC tumor tissue, we extract the Macrophages (*n* = 1175) from the single cell dataset (Fig. 1B). To determine whether the lncRNAs exert differential effects on HCC tumorigenesis, we compared the scRNA-seq transcriptome of each macrophage between tumor tissue and normal tissue. Among the differentially expressed transcripts, we observed that the RP11-1100 L3.8 (ENSG00000259884) is most significantly expressed in the tumor tissue with the high the effect size (|Cohens’ d| > 0.1 and *p*-value < 0.05) (Fig. 1C). UMAP analysis shows that the lncRNA RP11-1100 L3.8 was mainly enriched in macrophages (Fig. 1D), especially in the macrophage derived from hepatocellular carcinoma tissue (Fig. 1E). To elucidate whether RP11-1100 L3.8 plays a role in HCC tumorigenesis without any bias, we conducted GSEA analysis employing TCGA RNA-sequencing data (TCGA LIHC, *n* = 424). GSEA Enrichment plots demonstrated that the gene signatures related to glycolysis (Fig. 1F) and M2 polarized pathways (Fig. 1G-H) were significantly enhanced in patients with high expression of RP11-1100 L3.8, but not in low expressing patients. These results indicated that lncRNA RP11-1100 L3.8, which may mediate M2 macrophage polarization, mainly expressed in the macrophage cells of HCC patients. Therefore, we next focus on RP11-1100 L3.8 for further investigation and named it as lncMMPA (M2 macrophage polarization associated lncRNA).

### lncMMPA induces M2 macrophage polarization in HCC

**Fig. 2:**
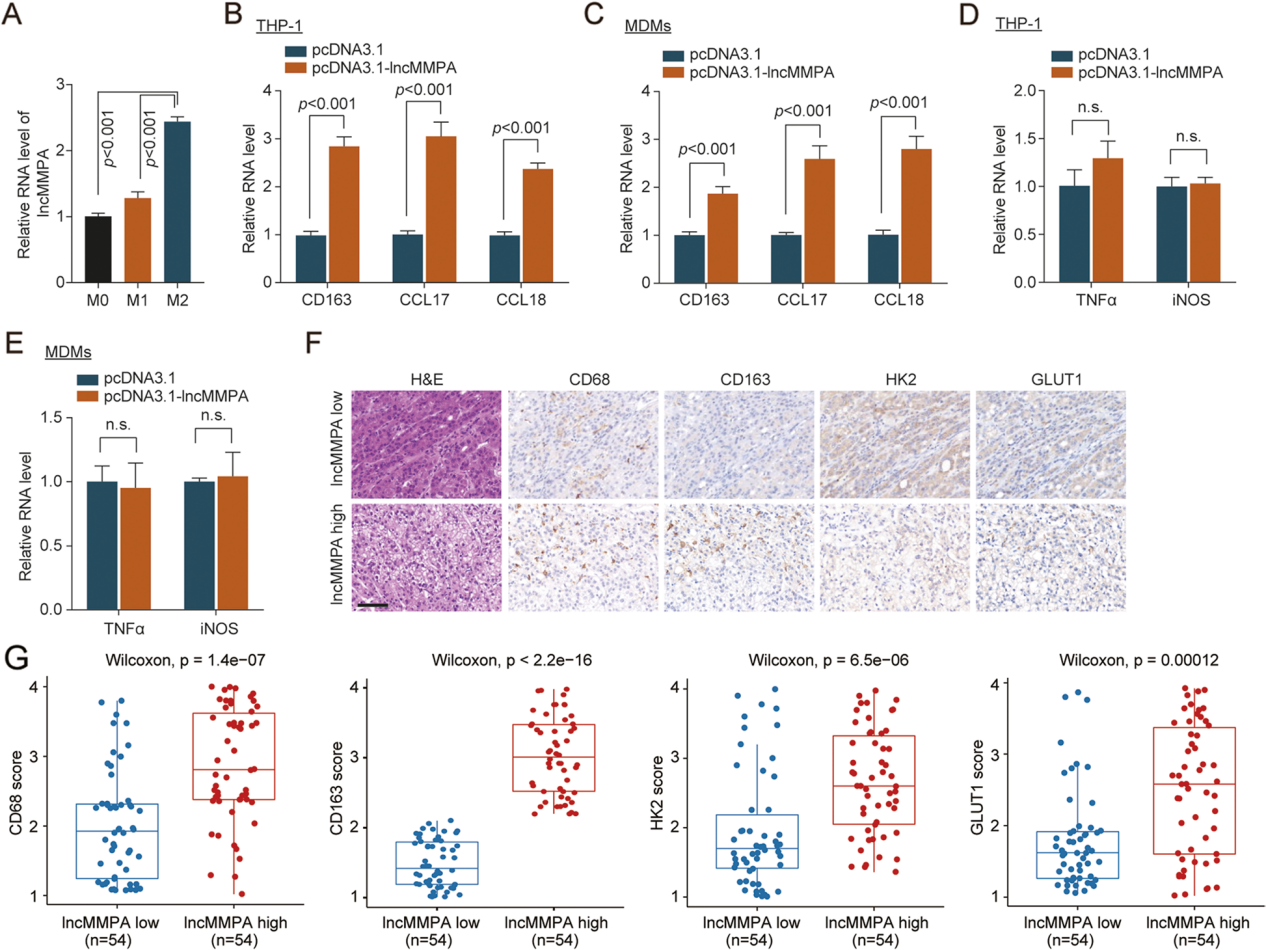
RP11-1100 L3.8 lncRNA induces M2 macrophage polarization. **A** The expression level of LncMMPA in M0, M1 and M2 macrophages derived from monocyte cells was normalized to that of actin gene and shown as a ratio relative to the expression level in the M0 macrophages. **B** The expression level of M2 macrophages markers (CD163, CCL17 and CCL18) in THP-1 cells with lncMMPA overexpression was normalized to that of actin gene and shown as a ratio relative to the expression level in THP-1 cells with pcDNA3.1 transfection. **C** The expression level of M2 macrophages markers (CD163, CCL17 and CCL18) in MDMs cells with lncMMPA overexpression was normalized to that of actin gene and shown as a ratio relative to the expression level in MDMs with pcDNA3.1 transfection. **D** The expression level of M1 macrophages markers (TNFα and iNOS) in THP-1 cells with lncMMPA overexpression was normalized to that of actin gene and shown as a ratio relative to the expression level in THP-1 cells with pcDNA3.1 transfection. **E** The expression level of M1 macrophages markers (TNFα and iNOS) in MDMs cells with lncMMPA overexpression was normalized to that of actin gene and shown as a ratio relative to the expression level in MDMs with pcDNA3.1 transfection. **F** Representative hematoxylin and eosin (H&E) and immunohistochemical (IHC) staining for CD68, CD163, GLUT1 and HK2 in HCC cancer samples with lncMMPA low or high expression level of ZSHS cohort 1; *n* = 108. Scale bar, 50 μm. **G** Boxplot show the IHC staining of CD68, CD163, GLUT1 and HK2 in lncMMPA low and high expression HCC tissue of ZSHS cohort 1; *n* = 108

To study the function of lncMMPA in M2 macrophage polarization, we evaluated lncMMPA expression, M1 markers and M2 markers in different human monocyte-derived macrophages (MDMs), which were separated from normal human peripheral blood monocyte cells. qRT-PCR data showed that lncMMPA expression gradually increased from M0, M1 then to M2 MDMs (Fig. 2A). The overexpression level of lncMMPA in THP-1 cells and human MDMs was showed in Supplementary Fig. S2A, B. Next evaluation revealed that overexpression of lncMMPA significantly increased the expression of M2 macrophage markers in THP-1 cells (Fig. 2B) and in MDMs (Fig. 2C). However, upregulation of lncMMPA did not significantly change the expression of M1 macrophage markers in THP-1 cells (Fig. 2D) and in MDMs (Fig. 2E). These data indicated that lncMMPA played an important role in the polarization of M2 macrophage. To assess the relationship between higher lncMMPA expression of TAMs and glycolysis of HCC, we conducted qRT-PCR analysis to measure lncMMPA (Supplementary Fig. S2C). In addition, immunostaining was done to examine the levels of CD68^+^ TAMs, and markers for glycolysis, including the glycolytic enzymes glucose transporter 1 (GLUT1) and hexokinase 2 (HK2). IHC staining analysis showed that GLUT1 and HK2, the key components of glycolysis, were significantly increased with abundant TAM infiltration (high level of CD68 and CD163) and high expression of lncMMPA in HCC tissues (ZSHS cohort 1, *n* = 108) (Fig. 2F). Quantification of the IHC score was done to confirm the positive correlation observed between lncMMPA and tumor glycolytic enzyme expression, including GLUT1 and HK2 or TAM infiltration (Fig. 2G). We further analyzed the correlation among lncMMPA expression level and different clinical presentations of pathophysiology of HCC in ZSHS cohort 1 (Supplementary Table S3).

### Human MDMs with higher lncMMPA augment aerobic glycolysis and reduce apoptosis of HCC cells

**Fig. 3:**
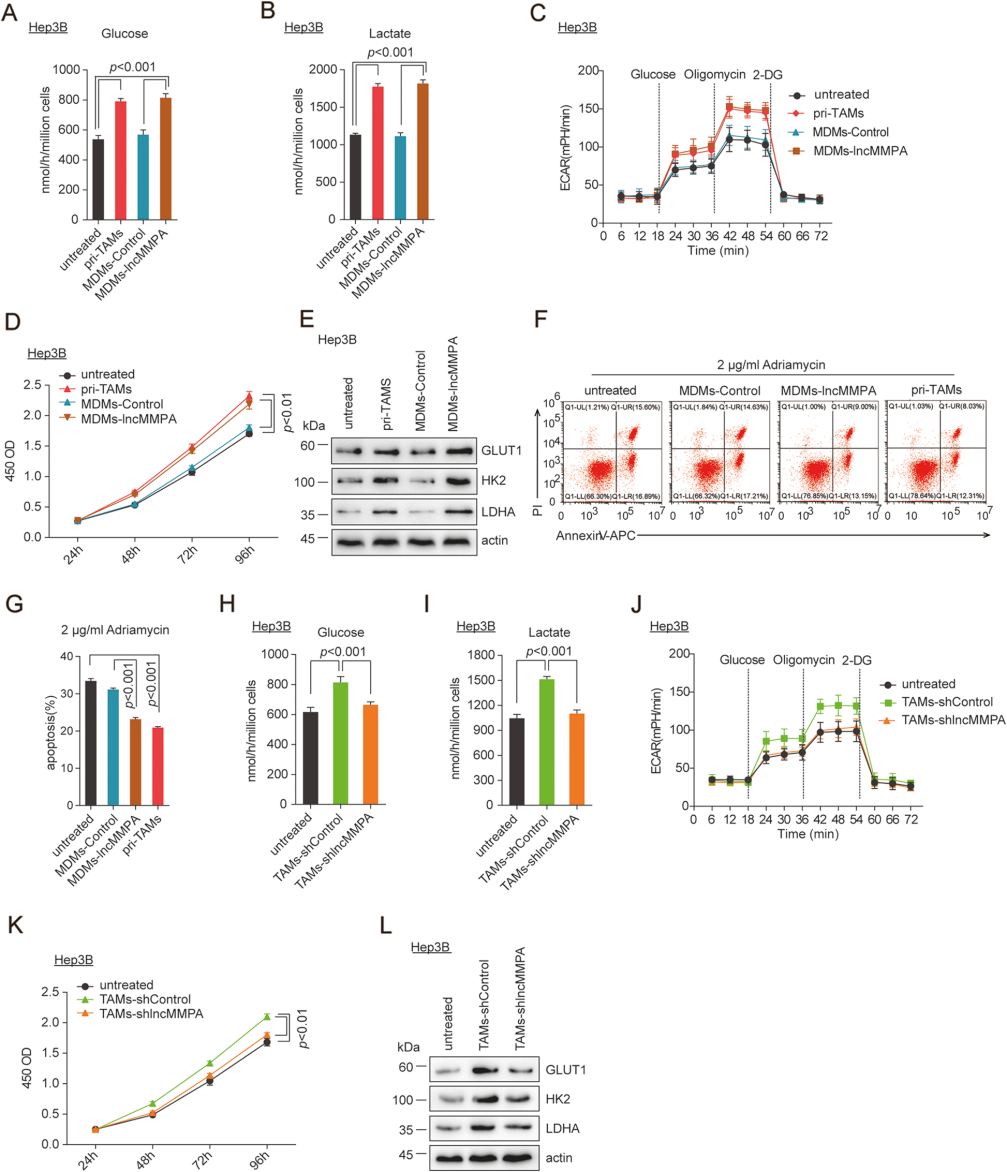
Human MDMs with higher lncMMPA increase aerobic glycolysis and decrease apoptosis of HCC cells. **A**-**G** Co-culture of Hep3B cells with pri-TAMs, MDMs-control or MDMs-lncMMPA was done in Transwell systems for 6 days. Then the Hep3B cells were harvested for the specified experiments. **A** Glucose consumption. **B** Lactate production. **C** ECAR. **D** Cell proliferation. **E** Expression of GLUT1, HK2 and LDHA. **F** The representative plots of flow cytometry analysis for Annexin V-APC and propidium iodide staining in Hep3B cells treated with Adriamycin (2 μg/ml) for 48 h. **G** The quantification of flow cytometry analysis in **F**. **H-L** Co-culture of Hep3B cells with TAMs-shControl or TAMs-shlncMMPA was done in Transwell systems for 6 days and then the Hep3B cells were harvested for the following experiments. **H** Glucose consumption. **I** Lactate production. **J** ECAR. **K** Cell proliferation. **L** Expression of GLUT1, HK2 and LDHA

Primary TAMs (pri-TAMs) from human HCC tissues (ZSHS cohort 2, *n* = 40) were purified to explore the role of human MDMs with higher lncMMPA expression in controlling tumor glycolysis. Primary TAMs were separated from fresh HCC tumor samples as described in Materials and methods. Then, the isolated TAMs and Hep3B HCC cells were co-cultured in a Transwell system, so that the TAMs and the HCC cells do not physically interact [22]. After 6 days inoculation, Hep3B cells co-cultured with pri-TAMs or MDMs with lncMMPA overexpression (lncMMPA-MDMs) exhibited higher consumption of glucose (Fig. 3A), lactate formation (Fig. 3B), extracellular acidification rates (ECARs) (Fig. 3C), and cell proliferation (Fig. 3D) in comparison to the cells cultured separately or after co-culture with MDMs. Besides, the expression of glycolytic enzymes, including GLUT1, HK2, and lactate dehydrogenase A (LDHA) was more in tumor cells after co-culture with TAMs (Fig. 3E). Similar enhanced glucose usage, lactate formation and cell proliferation were noticed in BEL-7404 cells following co-culture with pri-TAMs and lncMMPA-MDMs (Supplementary Fig. S3A-C). These data suggest that overexpression of lncMMPA enhanced aerobic glycolysis and cell proliferation in HCC cells. Moreover, co-culture of tumor cells with either pri-TAMs or lncMMPA-MDMs led to decreased apoptosis under 2 μg/ml Adriamycin chemotherapy (Fig. 3F-G). Thus, aerobic glycolysis in HCC cells, augmented by pri-TAMs and lncMMPA-MDMs, appears to confer resistance to apoptosis.

In loss of function assays, after co-culture with those TAMs with lncMMPA downregulation, higher glucose consumption (Fig. 3H), lactate production (Fig. 3I), ECARs (Fig. 3J), cell proliferation (Fig. 3K) and the expression of GLUT1, HK2 and LDHA (Fig. 3L) were effectively reduced in Hep3B cells, compared with those cultured with TAMs. The expression level of lncMMPA in TAMs with lncMMPA downregulation was showed in Supplementary Fig. S3D. These data indicated that lncMMPA promoted TAM-mediated activation of glycolysis in HCC cells.

### Exosomal lncMMPA enhances macrophage-induced tumor progression

**Fig. 4:**
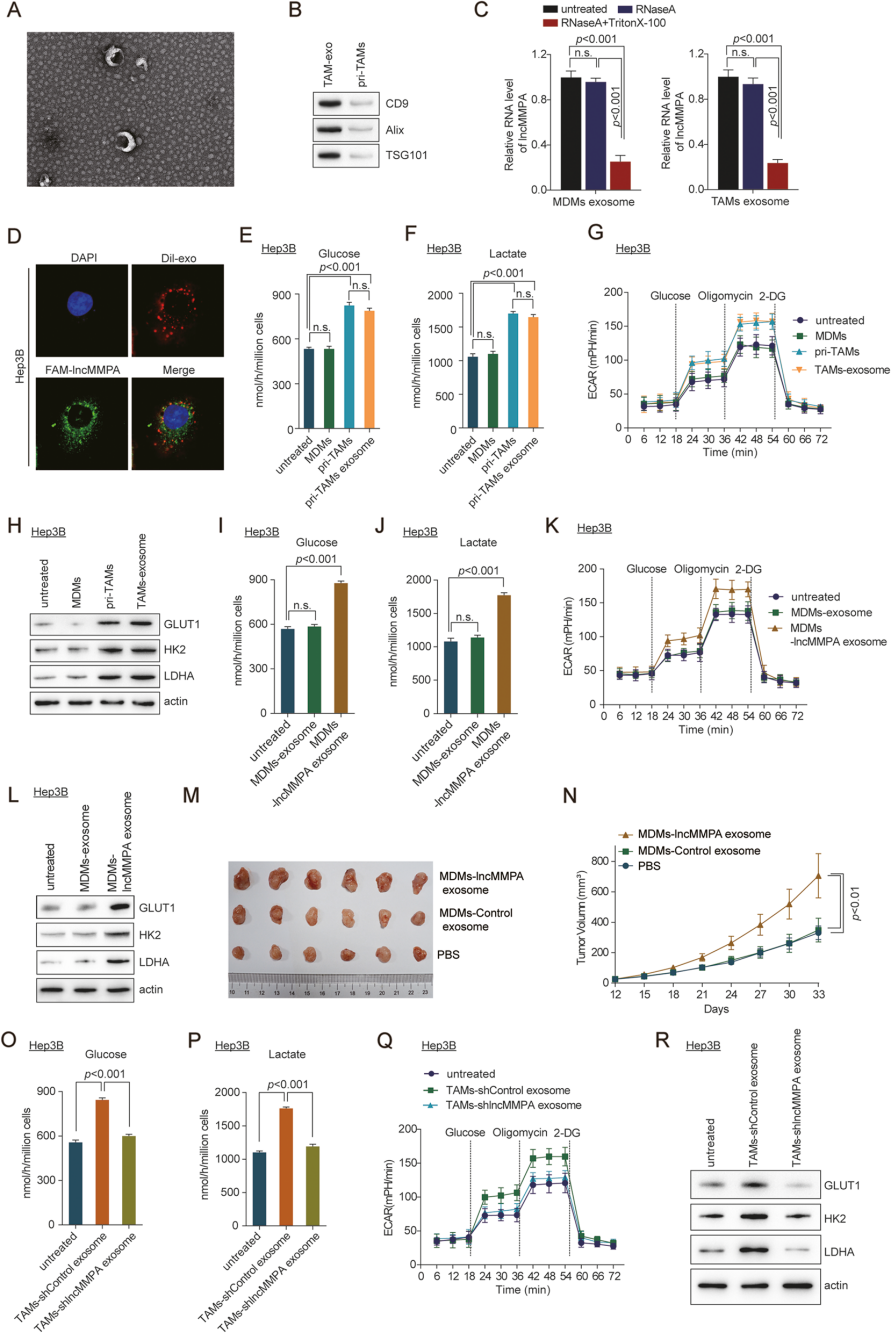
Exosomal lncMMPA enhances macrophage-induced tumor progression. **A** Electron microscope observation of the morphology of TAMs-derived exosomes. Scale bar, 100 nm. **B** Western blot analysis of antigens (CD9, Alix and TSG101) in TAMs-derived exosomes (TAMs-exo). **C** The expression level of lncMMPA in the equal numbers of exosomes derived from MDMs (left) and TAMs (right) after treatment with RNase A alone or together with Triton X-100 was normalized to that of cel-miR-39-3p and shown as a ratio relative to the expression level in untreated exosomes derived from MDMs or TAMs. **D** Fluorescent staining was conducted in Hep3B cells following incubation with Dil-labeled (red) exosomes obtained from TAMs. Scale bar, 20 μm. **E-H** Co-culture of Hep3B cells with MDMs and pri-TAMs was done in Transwell systems for 6 d. Separately, Hep3B cells were treated with exosome from TAMs for 48 h. After these treatments, Hep3B cells were collected for the specified experiments. **E** Glucose consumption. **F** Lactate production. **G** ECAR. **H** Expression of GLUT1, HK2 and LDHA. **I-L** Hep3B cells were treated with exosomes from MDMs with or without overexpressing lncMMPA and then harvested for the specified experiments. **I** Glucose consumption. **J** Lactate production. **K** ECAR. **L** Expression of GLUT1, HK2 and LDHA. **M**, **N** Hep3B cells were injected orthotopically into the right flank of BALB/c nude mice. The mice were given intratumoral injection of the indicated exosomes from MDMs every 3 d. **M** Representative images of tumors in the xenografts. **N** Tumor growth curves. **O-R** Hep3B cells were treated with exosomes from TAMs with or without shlncMMPA transfection and then collected for the indicated experiments. **O** Glucose consumption. **P** Lactate production. **Q** ECAR. **R** Expression of GLUT1, HK2 and LDHA

Considering that TAMs mediated effects on the different functions of tumor cells, we next examined which component of the TAM culture media participates in lncMMPA-mediated HCC progression [23]. lncRNA usually was wrapped in the exosomes and performed its biological function [24]. We first isolated the exosomes from pri-TAMs by centrifugation. Electromicroscopy data showed that pri-TAMs-exosomes (TAMs-exo) were around with a diameter of about 40–140 nm (Fig. 4A). Western blot analysis showed that exosome markers CD9 [25], Alix and TSG101 were identified in TAMs-exo (Fig. 4B). The data indicated that TAMs-exo was successfully isolated. Next, we investigated whether lncMMPA existed in the extracellular vesicles of macrophage cells. qRT-PCR showed that lncMMPA level was significantly downregulated in MDMs-exo and TAMs-exo following simultaneous treatment with RNase A and Triton X-100 treatment, but seldom affected by exposure to RNase A only (Fig. 4C), suggesting that extracellular lncMMPA was mostly contained within the exosomes without being released directly. In immunofluorescence assay using confocal microscopy, co-localization of Dil-labeled exosomes (red) and FAM-labeled lncMMPA (green) was noticed in the HCC cells following incubation with exosomes, implying that the exosomes-mediated transfer of lncMMPA could occur between macrophage and Hep3B cells (Fig. 4D). Validation assay showed that the pellets of the TAM macrophages culture media, which contain exosomes, significantly enhanced the glucose consumption (Fig. 4E), lactate productions (Fig. 4F) and ECARs (Fig. 4G) of Hep3B cells. Furthermore, Western blot data revealed that the expression of the GLUT1, HK2 and LDHA was significantly increased in Hep3B cells after TAM-exosome treatment (Fig. 4H). In addition, TAMs-exosome also dramatically increased Hep3B cell proliferation (Supplementary Fig. S4A).

Next, MDMs-exo delivering oe-lncMMPA and TAMs-exo delivering shlncMMPA were co-cultured with Hep3B cells and functional assays were performed as well. MDMs-lncMMPA-exo treatment dramatically increased the glucose consumption (Fig. 4I), lactate productions (Fig. 4J), ECARs (Fig. 4K) and the GLUT1, HK2 and LDHA in Hep3B cells (Fig. 4L). In addition, MDMs-lncMMPA-exo dramatically increased HCC cell multiplication both in vitro (Supplementary Fig. S4B) and in vivo (Fig. 4M-N, Supplementary Fig. S4C, D).

On the contrary, Hep3B cells treated with TAMs-exo transferring shlncMMPA caused the opposite effect on Hep3B cells (Fig. 4O-R, Supplementary Fig. S4E). GW4869 treatment, which belongs the inhibitor for blocking exosome production, significantly reduced the glucose consumption (Supplementary Fig. S4F), lactate productions (Supplementary Fig. S4G), ECARs (Supplementary Fig. S4H) and cell proliferation (Supplementary Fig. S4I) in Hep3B cells. These results suggested that lncMMPA was taking part in the TAMs-exo induction of glycolysis and cell proliferation in HCC cells.

### lncMMPA interacts with miR-548 s in HCC cells

**Fig. 5:**
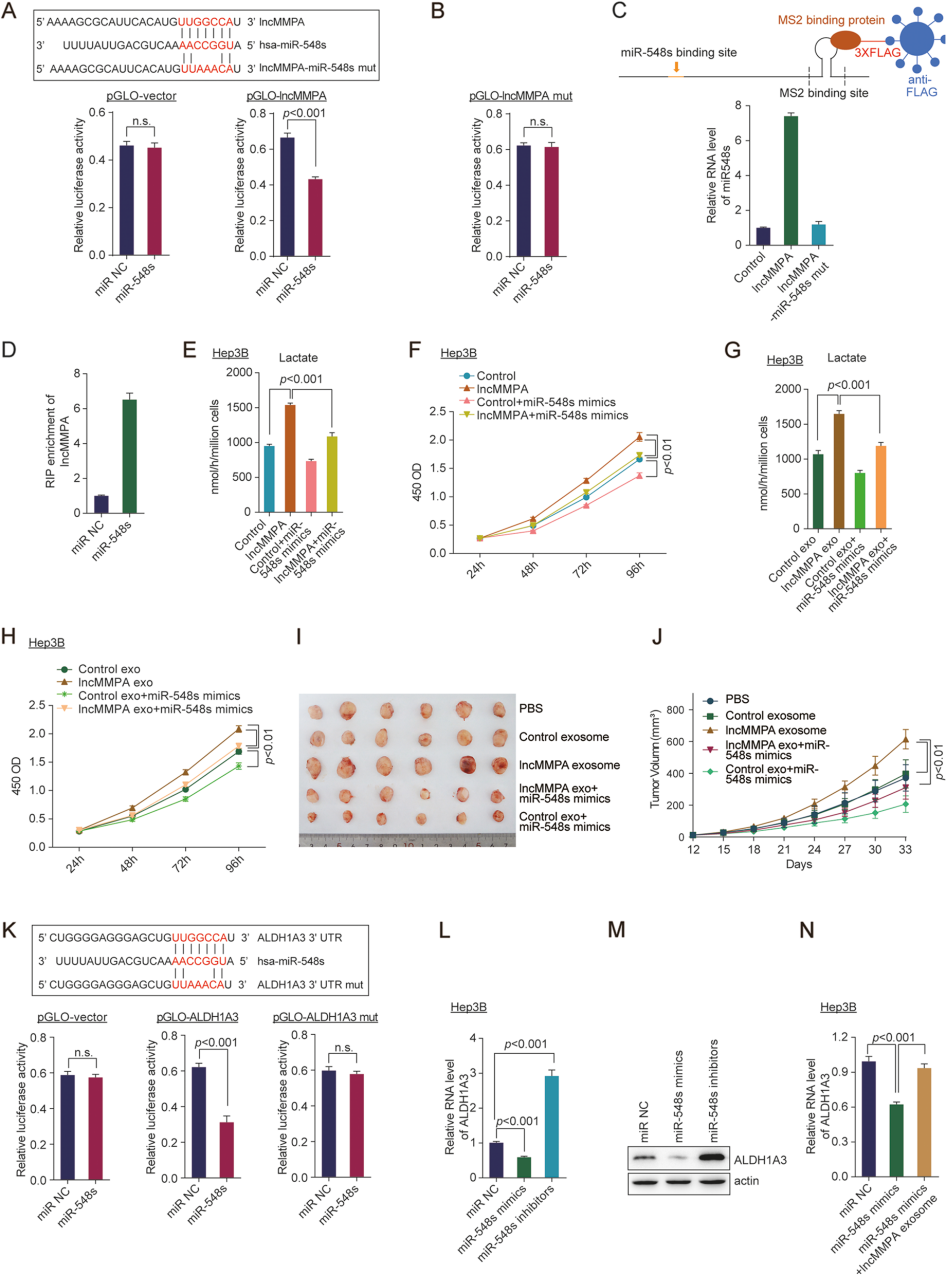
lncMMPA interacts with miR-548 s and mediates ALDH1A3 expression in HCC cells. **A** Sequence alignment of miR-548 s with lncMMPA and lncMMPA mutant (Top). Dual-luciferase reporter assays of pGLO-vector and pGLO-lncMMPA (bottom). **B** Dual-luciferase reporter assays of pGLO-lncMMPA mutant (pGLO-lncMMPA mut). **C** MS2-RIP followed by qRT-PCR to detect miR-548 s (lower panel) that endogenously related with lncMMPA. lncMMPA-miR-548 s mutant was employed as a negative control. RNA level of miR-548 s was shown as a ratio relative to the expression level in control sample. A schematic outline of the MS2-RIP strategy, which is employed for the identification of lncRNA associated endogenous microRNAs is shown (upper panel). **D** Anti-AGO2 RIP was conducted in Hep3B cells with transient overexpression of miR-548 s, and the expression level of lncMMPA associated with AGO2 was shown as a ratio relative to the expression level in miR NC transfected Hep3B cells. **E**, **F** Hep3B cells transfected with indicated microRNA mimics were co-cultured with MDMs-Control or MDMs-lncMMPA in Transwell systems for 6 days. These Hep3B cells were then harvested for the specified experiments. **E** Lactate production. **F** Cell proliferation. **G**, **H** Hep3B cells transfected with indicated microRNA mimics were treated with exosomes from MDMs-Control or MDMs-lncMMPA for 48 h and then harvested for the specified experiments. **G** Lactate production. **H** Cell proliferation. **I**, **J** Hep3B cells were injected orthotopically into the right flank of BALB/c nude mice (*n* = 6 per group). The mice were also given an intratumoral injection of the indicated exosomes from MDMs and miR-548 s mimics adenovirus every 3 d. **I** Representative images of tumors in the xenografts. **J** Tumor growth curves. **K** Sequence alignment of miR-548 s with ALDH1A3 and ALDH1A3 mutant (Top). Dual-luciferase reporter assays of pGLO-vector, pGLO-ALDH1A3 and pGLO-ALDH1A3 mutant (pGLO-ALDH1A3 mut) (bottom). **L** The RNA level of ALDH1A3 in Hep3B cells treated with the indicated microRNA inhibitors or mimics of miR-548 s was normalized to that of actin gene and shown as a ratio relative to the expression level in Hep3B cells with miR NC transfection. **M** The protein level of ALDH1A3 was detected in Hep3B cells treated with the indicated microRNA inhibitors or mimics of miR-548 s. **N** The RNA level of ALDH1A3 in Hep3B cells treated with the microRNA mimics of miR-548 s and lncMMPA as indicated was normalized to that of actin gene and shown as a ratio relative to the expression level in Hep3B cells with miR NC transfection

The potential mechanism of TAMs-exo lncMMPA in HCC cell glycolysis and proliferation was further explored. Accumulating evidence indicated that exosome wrapped lncRNA usually perform its biological function by acting as a ceRNA [24, 26]. Therefore, to study the influence of lncMMPA on the expression of miRNAs, the bioinformatics prediction analysis was performed by employing the Segal Lab program (Eran Segal; http://132.77.150.113/pubs/mir07/mir07_prediction.html) [27]. The analysis data revealed that miR-548 s is the potential candidate to bind lncMMPA (Fig. 5A).

To ascertain the association between miR-548 s and lncMMPA, we performed luciferase reporter assays utilizing the full sequence of lncMMPA (pGLO-lncMMPA). There was a significant reduction in the luciferase activity of the lncMMPA reporter plasmid by miR-548 s overexpression, but not the control plasmid (Fig. 5A). Furthermore, we constructed luciferase reporter plasmid with a mutated lncMMPA sequence that contained miR-548 s binding sites (pGLO-lncMMPA-mut). After transfecting these recombination plasmids into Hep3B cells, miR-548 s overexpression could not lower the luciferase activity of the lncMMPA-mut (Fig. 5B). To confirm the binding of lncMMPA with miR-548 s at endogenous levels, lncMMPA associated endogenous microRNAs were pulled down using MS2-RNA immunoprecipitation (MS2-RIP, Fig. 5, upper panel) [28]. This was done using an empty vector (MS2), a vector containing the full sequence of lncMMPA (designated lncMMPA) and a vector containing lncMMPA with mutations in the miR548s targeting binding sites [designated lncMMPA-mut (miR-548 s)]. Subsequent qRT-PCR analysis revealed that lncMMPA RIP was enriched significantly for miR-548 s in Hep3B cells compared with MS2 control and the lncMMPA mutated plasmid (Fig. 5C). MicroRNAs associate with their target mRNAs and lead to translational repression and/or RNA breakdown, which usually requires the AGO2 protein. To assess if lncMMPA binds to miR-548 s in this manner, anti-AGO2 RIP was conducted in Hep3B cells with transfection of miR-548 s mimics. RIP-qRT-PCR data showed that lncMMPA was elevated in cells transfected with miR-548 s mimics (Fig. 5D). These data indicate that lncMMPA may function as a ceRNA for miR-548 s in HCC cells.

In further functional validation, the lactate production (Fig. 5E, G) and cell proliferation (Fig. 5F, H) were significantly increased after MDMs-lncMMPA overexpression or MDMs-exo delivering oe-lncMMPA treatment in Hep3B cells, and tumor growth (Fig. 5I, J, Supplementary Fig. S5A-B) were significantly increased after MDMs-exo delivering oe-lncMMPA in mice tumor model. This increase was dramatically blocked after transfection of miR-548 s (Fig. 5E-J, Supplementary Fig. S5A-B). These data indicated that miR-548 s participates in lncMMPA-induced cell glycolysis and proliferation in HCC cells.

### MiR-548 s mediates lncMMPA-upregulated ALDH1A3 expression in HCC cells

Targetscan (http://www.targetscan.org/) [29] was employed to predict genes that share the regulatory role of miR-548 s with lncMMPA. After bioinformatical analysis (Supplementary Fig. S5C), ALDH1A3, which plays significant roles in tumorigenesis and glycolysis in various carcinogenesis [30, 31], was chosen for further validation. Next, luciferase assays were conducted and the data showed that transfection of miR-548 s mimics decreased the luciferase activity due to the ALDH1A3 reporter plasmid, but not the mutated plasmid (Fig. 5K). qRT-PCR (Fig. 5L) and Western blot assays (Fig. 5M) demonstrated that ALDH1A3 expression was lowered by miR-548 s mimics and elevated by miR-548 s inhibitors. Furthermore, overexpression of lncMMPA dramatically blocked miR-548 s-induced downregulation of ALDH1A3 in Hep3B cells (Fig. 5N). Knockdown of ALDH1A3 significantly blocked lncMMPA-induced increased in lactate production (Supplementary Fig. S5D) and Hep3B cell proliferation (Supplementary Fig. S5E). Overexpression of ALDH1A3 rescued miR-548 s mimics reduced lactate production (Supplementary Fig. S5F) and cell proliferation in Hep3B cells with lncMMPA overexpression (Supplementary Fig. S5G). These results suggested that ALDH1A3 is the direct target of miR-548 s in Hep3B cells, and lncMMPA-induced HCC cell glycolysis and proliferation depends on blocking miR-548 s-mediated.

### The expression of lncMMPA was associated with glycolysis components in HCC patients

**Fig. 6:**
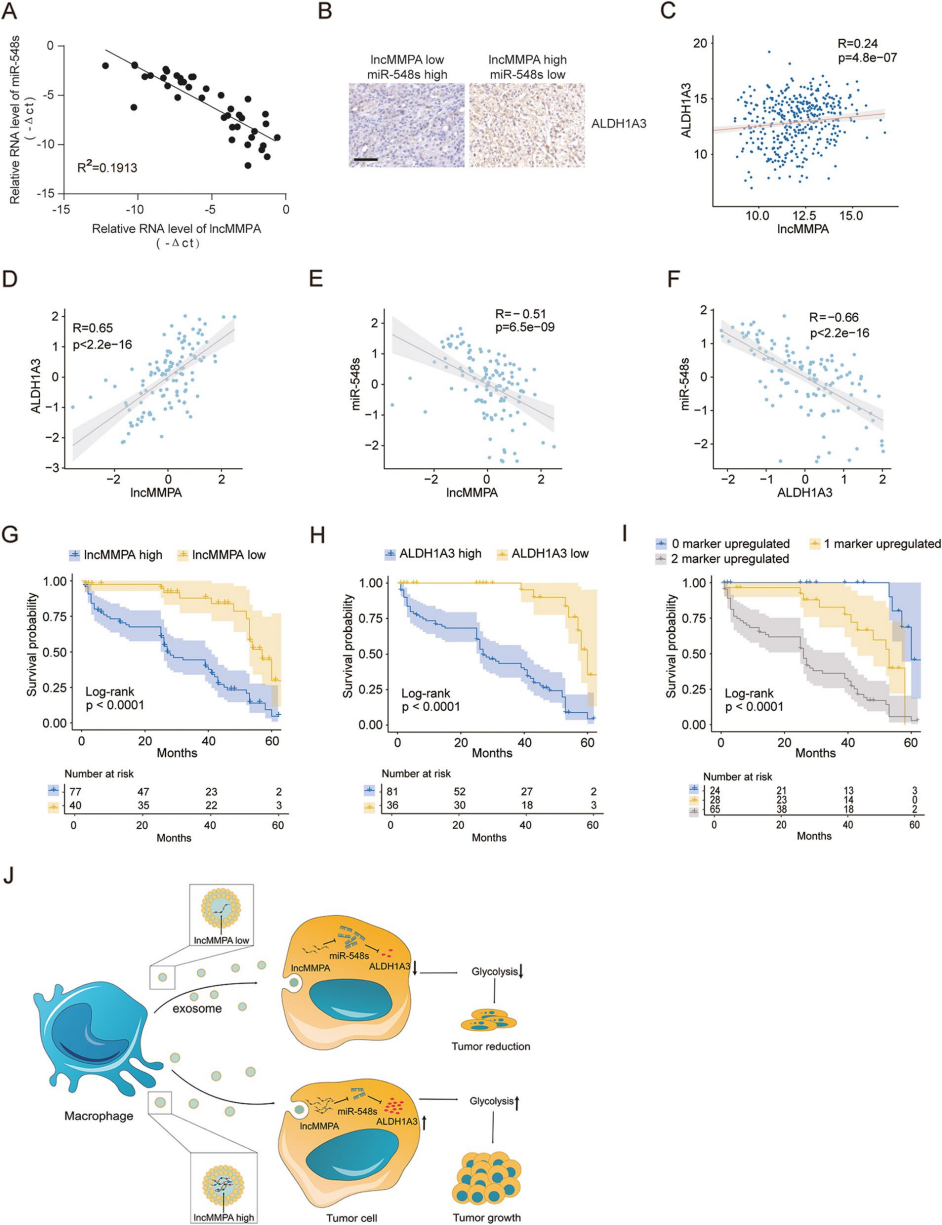
The expression and correlation analysis between lncMMPA and glycolysis components in CRC patients. **A** Correlation analysis between lncMMPA expression and miR-548 s expression in HCC tissues of ZSHS cohort 2; *n* = 40. **B** Representative immunohistochemical (IHC) staining for ALDH1A3 in HCC cancer samples with lncMMPA low and miR-548 s high or lncMMPA high and miR-548 s low expression level of ZSHS cohort 1; *n* = 108. Scale bar, 50 μm. **C** Correlation analysis between lncMMPA expression and ALDH1A3 in HCC tissues, *n* = 424, in TCGA LIHC dataset. **D** Correlation analysis between lncMMPA expression and ALDH1A3 expression in HCC tissues of ZSHS cohort 3; *n* = 117. **E** Correlation analysis between lncMMPA expression and miR548s expression in HCC tissues of ZSHS cohort 3; *n* = 117. **F** Correlation analysis between ALDH1A3 expression and miR548s expression in HCC tissues of ZSHS cohort 3; *n* = 117. **G** Survival was determined and compared between patients showing high and low levels of lncMMPA expression in the tumor, in ZSHS cohort 3; *n* = 117, log-rank test. **H** Survival was assessed and compared between patients showing high and low levels of ALDH1A3 expression in the tumor in ZSHS cohort 3; *n* = 117, log-rank test. **I** Survival was analyzed and compared among HCC patients based on the number of upregulated molecular markers (lncMMPA and ALDH1A3) in ZSHS cohort 3; *n* = 117. **J** Potential schematic pathway illustrated the role of lncMMPA in HCC progression

Next qRT-PCR assay was done to examine the expression of lncMMPA, miR-548 s, and immunohistochemical staining assay was done to assess ALDH1A3 expression in HCC patient’ tissues of ZSHS cohort. Correlation analysis revealed that miR-548 s expression negatively related with lncMMPA in HCC tissues of ZSHS cohort 2 (Fig. 6A). Cancer tissues of HCC patients with higher expression of lncMMPA displayed higher levels of ALDH1A3, and vice versa, in ZSHS cohort 1 (Fig. 6B) and TCGA LIHC dataset (Fig. 6C). Further validation assay showed that lncMMPA expression is positively correlated with ALDH1A3 expression (Fig. 6D), and negatively correlated with miR-548 s (Fig. 6E) in HCC tissues of ZSHS cohort 3. In addition, ALDH1A3 expression negatively correlated with miR-548 s as well in HCC tissues of ZSHS cohort 3 (Fig. 6F). We next explored the correlation of overall survival in this HCC patient’s cohort by evaluating the intensity of lncMMPA and ALDH1A3. The shortest overall survival times were noticed in those patients with increased lncMMPA and ALDH1A3 expression in ZSHS cohort 3 (Fig. 6G-I). These data indicated that higher expression levels of lncMMPA and its target genes may identify HCC patients with poor prognosis.

## Discussion

Various etiological factors including non-alcoholic steatohepatitis, viral infections causing hepatitis, and excessive alcohol consumption are known to the development of HCC, which belongs an inflammation-associated cancer [32, 33]. In this study, we identified the biological function of lncMMPA from TAMs-exo in the polarization of macrophage and the process of glycolysis pathway activation and cell proliferation in HCC (Fig. 6J). Through statistical analysis, lncMMPA, which is derived from TAM, was discovered to induce M2 macrophage polarization. Then, the level of lncMMPA was higher in HCC and this lncRNA triggered HCC cells to enhance glucose metabolism and accelerated HCC cell proliferation. In combination, TAMs-exo could deliver lncMMPA to promote HCC tumor growth in vivo through stimulating glycolysis pathway. Finally, lncMMPA could interact with miR-548 s to target ALDH1A3, and then further regulated cell glucose metabolism and facilitated cell proliferation in HCC. Our results suggest lncMMPA blockade is a potential novel approach for the treatment of HCC.

Exosomes are usually constituted by small vesicles that are released outside the cell, and these are generally from multivesicular bodies of late endosomes [34, 35]. The functions of TAMs-exo have been widely investigated in various types of human cancer [12]. It has recently been shown that exosomes from macrophages contain a cargo of miRNAs that are used for inter-cellular communication, which is a contributory factor to the development of many diseases [36]. MiR-183-5p is enriched in exosomes arising from M2-polarized macrophages. M2-TAMs-exo could transfer miR-183-5p into gastric cancer cells and further promote the malignant phenotype of this cancer [37]. In addition, miR-223, which is secreted by IL-4-activated macrophages, could be delivered to breast cancer cells, and further facilitate breast cancer cell invasion, which is mediated by TAMs [38]. LncRNAs have been extensively explored to modulate the polarization of macrophages and to mediate the malignant behavior in HCC cells [39]. In this study, we firstly reveal that TAMs-derived exosomes could deliver lncRNAs and modulate the biological function of HCC cells.

As an important hallmark in carcinogenesis, metabolic reprogramming usually is regulated by the microenvironment. Amino acid metabolism can be regulated by effector T cells, which further affect the chemo-resistance in ovarian cancer [40]. Lactate could mediate the activation of TAMs and further promote breast cancer progression [41]. However, the mechanism by which TAMs modulate tumor glycolysis and progression is yet to be fully understood. Our current study has demonstrated that TAMs of HCC generate exosomes to transmit lncMMPA, instead of cytokines or microRNA, to increase aerobic glycolysis and proliferation in HCC cells. Exosomes contain numerous noncoding RNAs, including microRNAs and lncRNAs [42]. Exosomes-wrapped small nuclear RNAs and microRNAs can be effectively transported to target cells to control their biological function [43, 44]. Here, lncMMPA, a myeloid-specific lncRNA, is released via exosomes from TAMs to tumor cells to stabilize ALDH1A3 and augment proliferation and aerobic glycolysis in HCC cells. lncMMPA (RP11-1100 L3.8) was mainly expressed in the TAM and malignant cells, and the lncMMPA expression in HCC cells is much lower than that in TAM [45]. Moreover, lncRNAs can exhibit biological effects only when they reach a certain abundance [46]. These data indicated that lncMMPA is probably from TAM and drives HCC progression.

Currently, the cancer therapeutic targets have move to metabolic reprogramming of cancer cells [47]. Here, we showed that interfering with a TAM-specific lncMMPA decreases glycolysis and cell proliferation in HCC cells, emphasizing that lncMMPA is a promising therapeutic candidate for HCC. Even though small-molecule compounds that inhibit glycolytic enzymes have been suggested as potential therapeutics for HCC, several concerns such as lack of specific effects of these drugs on tumor enzymes vs normal cell enzymes, are yet to be resolved. In addition, even if cancer-specific isoforms are inhibited selectively, this may cause induction of the expression of alternative isoforms, resulting in drug resistance [47]. Here, our results have revealed that lncMMPA-mediated the stabilization of ALDH1A3, which may offer a more promising method to block glycolysis in HCC cells.

## Conclusion

In summary, our study delineates the mechanisms of macrophage-mediated regulation of the metabolic reprogramming in HCC cells via the exosomal delivery of oncogenic lncMMPA and the significance of cell-cell interactions in this process. Although more evidence is still needed, our study provides a novel target for the treatment of HCC.

## Supplementary Information

**Additional file 1: Supplementary Table S1.** The sequences of microRNA mimics, microRNA inhibitors, and shRNA used in the study. **Supplementary Table S2.** Primer sequences used in the study. **Supplementary Table S3.** Clinicopathological characteristics and follow-up data of 108 patients with HCC. **Supplementary Fig. S1.** RP11-1100 L3.8 lncRNA is clinically relevant in hepatocellular carcinoma. UMAP plot of all cells colored by each donor (GSE140228). **Supplementary Fig. S2.** lncMMPA induces M2 macrophage polarization in HCC. A. The relative lncMMPA expression level in lncMMPA overexpressing THP1 was normalized to that of actin gene and shown as a ratio relative to the expression level in mock cells. B. The relative lncMMPA expression level in lncMMPA overexpressing MDMs was normalized to that of actin gene and shown as a ratio relative to the expression level in mock cells. C. The relative lncMMPA expression level in tissue samples, and samples were divided into lncMMPA low and lncMMPA high group according to lncMMPA expression. **Supplementary Fig. S3.** Human MDMs with higher lncMMPA elevate aerobic glycolysis and lower apoptosis of HCC cells. A-C. Co-culture of BEL-7404 cells was done in Transwell systems with pri-TAMs, MDMs-control or MDMs-lncMMPA for 6 days. Then the BEL-7404 cells were harvested for the specified experiments. A. Glucose consumption. B. Lactate production. C. Cell proliferation. D. The relative lncMMPA expression level in TAMs transduced with indicated shlncMMPAs. **Supplementary Fig. S4.** Exosomal lncMMPA enhances macrophage-induced tumor progression. A. Hep3B cells were co-cultured with MDMs and pri-TAMs in Transwell systems for 6 days or treated with exosome from TAMs and then cell proliferation was performed by CCK8 assay. B. Hep3B cells were treated with exosome from MDMs with or without overexpressing lncMMPA for 48 h and then cell proliferation was performed by CCK8 assay. C. Ki-67 staining was performed after exosomes from MDMs-lncMMPA treatments in the xenograft mouse model. Scale bar, 50 μm. D. The quantification of Ki-67 staining in Supplementary Fig. 4C. E. Hep3B cells were treated with exosomes from TAMs-shControl or TAMs-shlncMMPA for 48 h and then cell proliferation was performed by CCK8 assay. F-I. Hep3B cells were treated with exosomes from TAMs with or without GW4869 treatment and then collected for the indicated experiments. F. Glucose consumption. G. Lactate production. H. ECAR. I. Cell proliferation. **Supplementary Fig. S5.** lncMMPA interacts with miR-548 s and mediates ALDH1A3 expression in HCC cells. A. Ki-67 staining was performed after exosomes from MDMs-lncMMPA and miR548s mimics treatments in the xenograft mouse model. Scale bar, 50 μm. B. The quantification of Ki-67 staining in Supplementary Fig. 5A. C. The flow chart for selected candidate target gene of lncMMPA and miRNA-548 s is shown. D, E. Hep3B cells transfected with indicated siRNAs were co-cultured with MDMs-Control or MDMs-lncMMPA in Transwell systems for 6 d and then collected for the indicated experiments. D. Lactate production of Hep3B cells with indicated treatments. E. Cell proliferation of Hep3B cells with indicated treatments. F, G. Hep3B cells transfected with indicated microRNA mimics and ALDH1A3 overexpression plasmid were co-cultured with MDMs-Control or MDMs-lncMMPA in Transwell systems for 6 d and then collected for the indicated experiments. F. Lactate production of Hep3B cells with indicated treatments. G. Cell proliferation of Hep3B cells with indicated treatments.

## Data Availability

The datasets used and/or analyzed during the current study are available from the corresponding author on reasonable request.

## Abbreviations

HCC: Hepatocellular carcinoma
TAM: Tumor-associated macrophage
lncRNAs: Long noncoding RNAs
ECAR: Extracellular acidification rates
lncMMPA: M2 macrophage polarization associated lncRNA
TME: Tumor microenvironment
H&E: Hematoxylin and eosin
IHC: Immunohistochemistry
GLUT1: Glucose transporter 1
HK2: Hexokinase 2
LDHA: Lactate dehydrogenase A
ZSHS: Zhongshan Hospital
