# Supplementary material for: Tumor associated macrophages-derived exosomes facilitate hepatocellular carcinoma malignance by transferring lncMMPA to tumor cells and activating glycolysis pathway
Source: J Exp Clin Cancer Res. 2022 Aug 19;41:253. doi: 10.1186/s13046-022-02458-3 (PMC9389814; doi:10.1186/s13046-022-02458-3)
Supplement: Supplementary file 1 — Additional file 1: Supplementary Table S1. The sequences of microRNA mimics, microRNA inhibitors, and shRNA used in the study. Supplementary Table S2. Primer sequences used in the study. Supplementary Table S3. Clinicopathological characteristics and follow-up data of 108 patients with HCC. Supplementary Fig. S1. RP11-1100 L3.8 lncRNA is clinically relevant in hepatocellular carcinoma. UMAP plot of all cells colored by each donor (GSE140228). Supplementary Fig. S2. lncMMPA induces M2 macrophage polarization in HCC. A. The relative lncMMPA expression level in lncMMPA overexpressing THP1 was normalized to that of actin gene and shown as a ratio relative to the expression level in mock cells. B. The relative lncMMPA expression level in lncMMPA overexpressing MDMs was normalized to that of actin gene and shown as a ratio relative to the expression level in mock cells. C. The relative lncMMPA expression level in tissue samples, and samples were divided into lncMMPA low and lncMMPA high group according to lncMMPA expression. Supplementary Fig. S3. Human MDMs with higher lncMMPA elevate aerobic glycolysis and lower apoptosis of HCC cells. A-C. Co-culture of BEL-7404 cells was done in Transwell systems with pri-TAMs, MDMs-control or MDMs-lncMMPA for 6 days. Then the BEL-7404 cells were harvested for the specified experiments. A. Glucose consumption. B. Lactate production. C. Cell proliferation. D. The relative lncMMPA expression level in TAMs transduced with indicated shlncMMPAs. Supplementary Fig. S4. Exosomal lncMMPA enhances macrophage-induced tumor progression. A. Hep3B cells were co-cultured with MDMs and pri-TAMs in Transwell systems for 6 days or treated with exosome from TAMs and then cell proliferation was performed by CCK8 assay. B. Hep3B cells were treated with exosome from MDMs with or without overexpressing lncMMPA for 48 h and then cell proliferation was performed by CCK8 assay. C. Ki-67 staining was performed after exosomes from MDMs-lncMMPA treatments in the [file 13046_2022_2458_MOESM1_ESM.docx]

**Supplementary Table S1. The sequences of microRNA mimics, microRNA inhibitors, and shRNA used in the study**

| shRNA_id | Sequence (5’-3’) |
| --- | --- |
| miR NC | CAAAGAAGCAAGAAUUCAAGA |
| miR-548s mimic | ATGGCCAAAACTGCAGTTATTTT |
| miR-548s inhibitor | AAAATAACTGCAGTTTTGGCCAT |
| shControl | ACCACGCTCTTTATCTTTG |
| shlncMMPA-1 | CAAGCTCTTTCTTCCTGTA |
| shlncMMPA-2 | GCACGGCGCTATGTGTTAA |

**Supplementary Table S2. Primer sequences used in the study**

| Gene | Forward/Reverse | Sequence (5’-3’) |
| --- | --- | --- |
| lncMMPA | Forward | CTCCCACCTCAAGCTCTTT |
|  | Reverse | CCCACGATTTGTCTTATCCC |
| CD163 | Forward | TTTGTCAACTTGAGTCCCTTCAC |
|  | Reverse | TCCCGCTACACTTGTTTTCAC |
| CCL17 | Forward | TCTTGAAGCCTCCTCACC |
|  | Reverse | CACTGTGGCTCTTCTTCGTC |
| CCL18 | Forward | CCAGTGAACTTGGTGGGC |
|  | Reverse | GGCATAGCAGATGGGACT |
| TNF-α | Forward | CCTCTCTCTAATCAGCCCTCTG |
|  | Reverse | GAGGACCTGGGAGTAGATGAG |
| iNOS | Forward | AGCATCCACGCCAAGAAC |
|  | Reverse | TCCACAACTCGCTCCAAG |
| ALDH1A3 | Forward | TGAATGGCACGAATCCAAGAG |
|  | Reverse | CACGTCGGGCTTATCTCCT |
| β-actin | Forward | GATGACCCAGATCATGTTTGAG |
|  | Reverse | TAATGTCACGCACGATTTCC |
| miR-548s | Forward | AACAAGATGGCCAAAACTGCAGT |
|  | Reverse | CAGTGCAGGGTCCGAGGT |
| U6 | Forward | AAAGCAAATCATCGGACGACC |
|  | Reverse | GTACAACACATTGTTTCCTCGGA |

**Supplementary Table S3. Clinicopathological characteristics and follow-up data of 108 patients with HCC**

| Characteristics | lncMMPA expression | | *P* value |
| --- | --- | --- | --- |
|  | Low | High |  |
| Gender |  |  | 0.111 |
| Male (n=68) | 38 | 30 |  |
| Female (n=40) | 16 | 24 |  |
| Age (years) |  |  | 0.240 |
| < 55 (n=44) | 19 | 25 |  |
| ≥ 55 (n=64) | 35 | 29 |  |
| AFP (ng/mL) |  |  | 0.123 |
| > 200 (n=52) | 30 | 22 |  |
| ≤ 200 (n=56) | 24 | 32 |  |
| TNM stage |  |  | 0.012 |
| I+ II (n=47) | 30 | 17 |  |
| III (n=61) | 24 | 37 |  |
| HBV* infection  Yes (n=68)  No (n=40) | 26  28 | 42  12 | 0.001 |

*HBV, hepatitis B virus.

Differences between groups were determined by the Chi-square test.

**
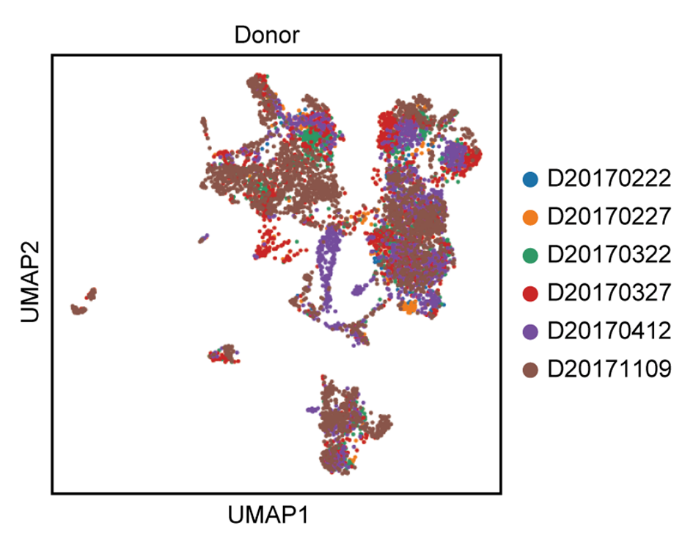
**

**Supplementary Figure S1. RP11-1100L3.8 lncRNA is clinically relevant in hepatocellular carcinoma**

UMAP plot of all cells colored by each donor (GSE140228).

**
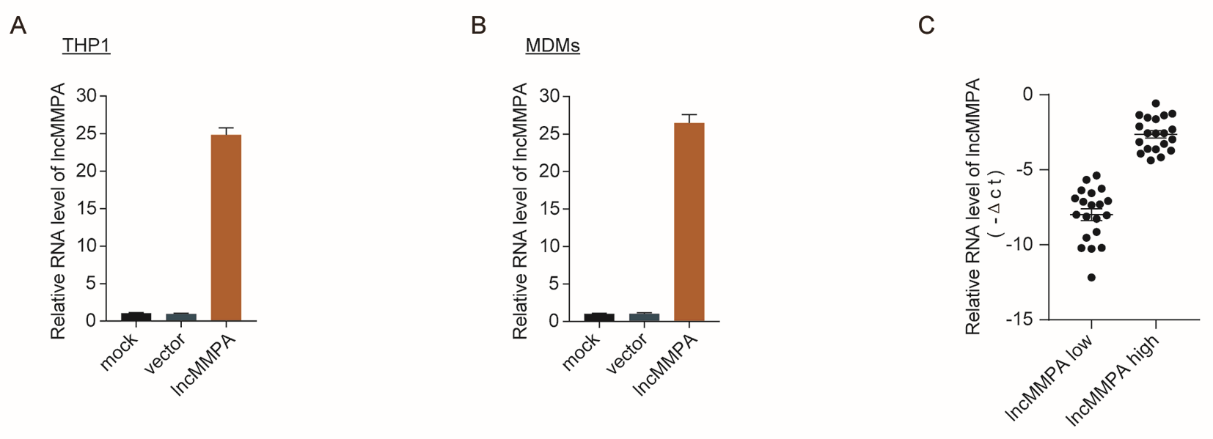
**

**Supplementary Figure S2. lncMMPA induces M2 macrophage polarization in HCC**

**A.** The relative lncMMPA expression level in lncMMPA overexpressing THP1 was normalized to that of actin gene and shown as a ratio relative to the expression level in mock cells.

**B.** The relative lncMMPA expression level in lncMMPA overexpressing MDMs was normalized to that of actin gene and shown as a ratio relative to the expression level in mock cells.

**C.** The relative lncMMPA expression level in tissue samples, and samples were divided into lncMMPA low and lncMMPA high group according to lncMMPA expression.

**
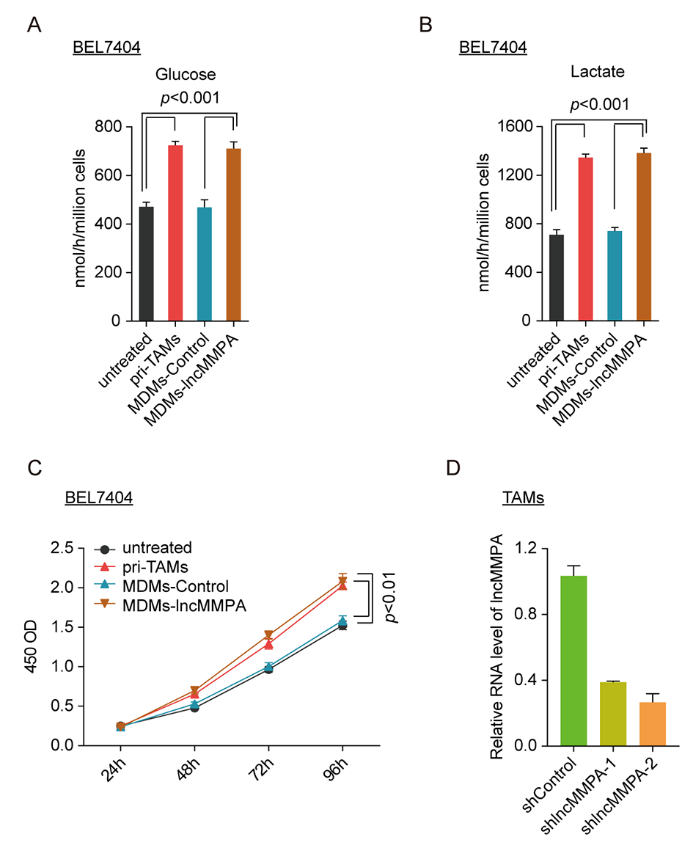
**

**Supplementary Figure S3. Human MDMs with higher lncMMPA elevate aerobic glycolysis and lower apoptosis of HCC cells**

**A-C.** Co-culture of BEL-7404 cells was done in Transwell systems with pri-TAMs, MDMs-control or MDMs-lncMMPA for 6 days. Then the BEL-7404 cells were harvested for the specified experiments. **A.** Glucose consumption. **B.** Lactate production. **C.** Cell proliferation.

**D.** The relative lncMMPA expression level in TAMs transduced with indicated shlncMMPAs.

**
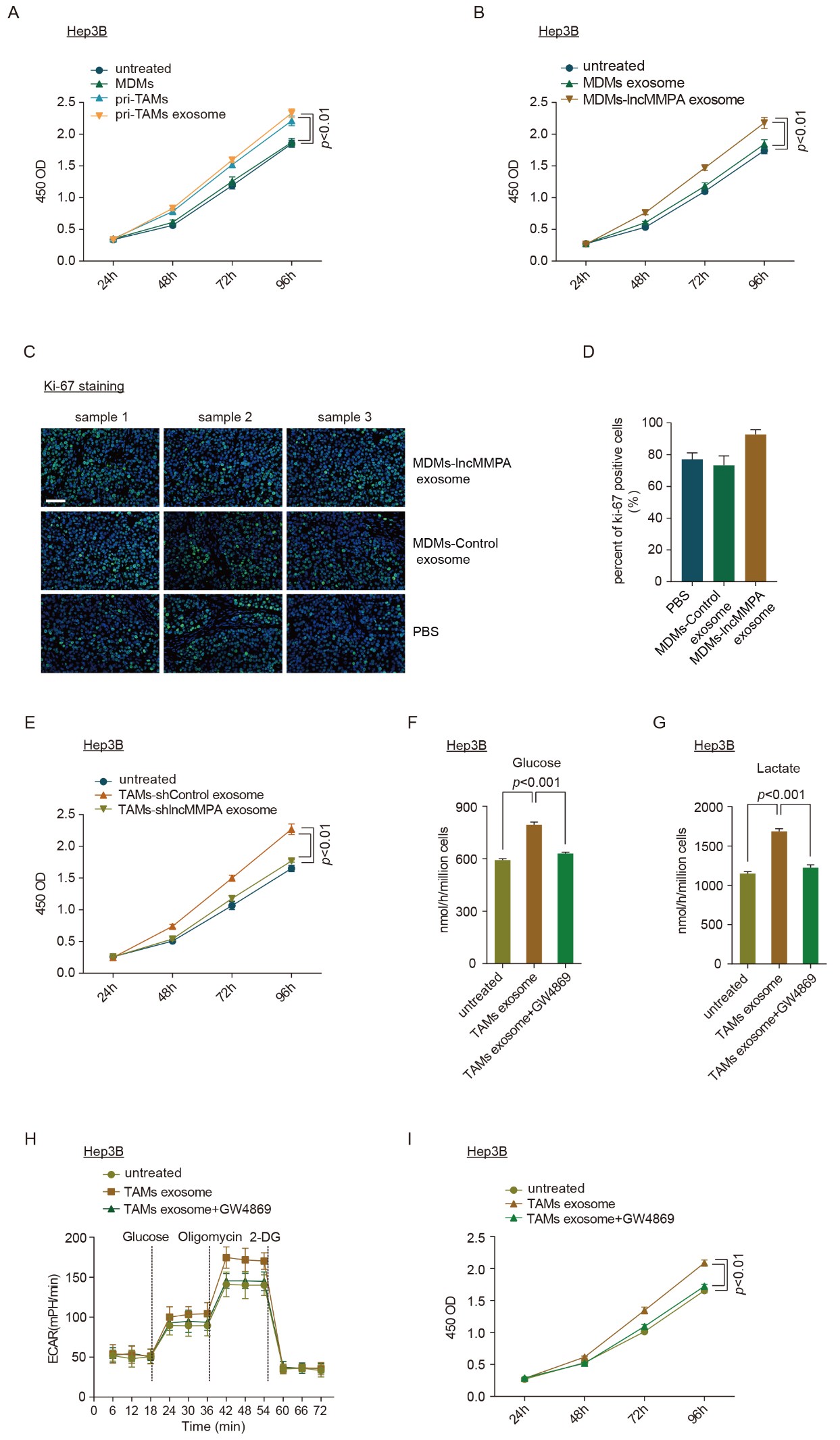
**

**Supplementary Figure S4. Exosomal lncMMPA enhances macrophage-induced tumor progression**

**A.** Hep3B cells were co-cultured with MDMs and pri-TAMs in Transwell systems for 6 days or treated with exosome from TAMs and then cell proliferation was performed by CCK8 assay.

**B.** Hep3B cells were treated with exosome from MDMs with or without overexpressing lncMMPA for 48 h and then cell proliferation was performed by CCK8 assay.

**C.** Ki-67 staining was performed after exosomes from MDMs-lncMMPA treatments in the xenograft mouse model. Scale bar, 50 μm.

**D.** The quantification of Ki-67 staining in Supplementary Figure 4C.

**E**. Hep3B cells were treated with exosomes from TAMs-shControl or TAMs-shlncMMPA for 48 h and then cell proliferation was performed by CCK8 assay.

**F-I.** Hep3B cells were treated with exosomes from TAMs with or without GW4869 treatment and then collected for the indicated experiments. **F.** Glucose consumption. **G.** Lactate production. **H.** ECAR. **I.** Cell proliferation.

**
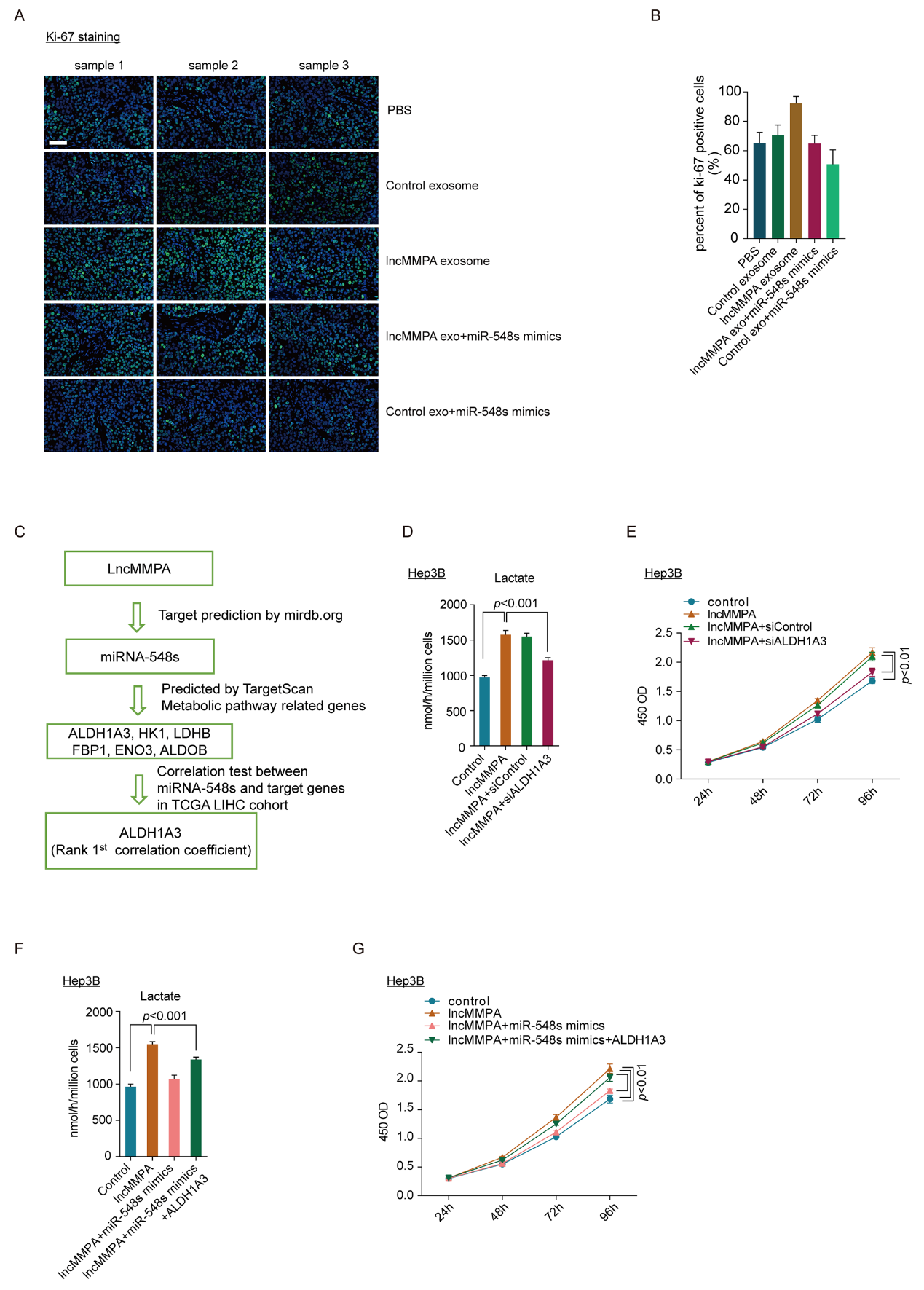
**

**Supplementary Figure S5. lncMMPA interacts with miR-548s and mediates ALDH1A3 expression in HCC cells**

**A.** Ki-67 staining was performed after exosomes from MDMs-lncMMPA and miR548s mimics treatments in the xenograft mouse model. Scale bar, 50 μm.

**B.** The quantification of Ki-67 staining in Supplementary Figure 5A.

**C.** The flow chart for selected candidate target gene of lncMMPA and miRNA-548s is shown.

**D, E.** Hep3B cells transfected with indicated siRNAs were co-cultured with MDMs-Control or MDMs-lncMMPA in Transwell systems for 6 d and then collected for the indicated experiments. **D.** Lactate production of Hep3B cells with indicated treatments. **E.** Cell proliferation of Hep3B cells with indicated treatments.

**F, G.** Hep3B cells transfected with indicated microRNA mimics and ALDH1A3 overexpression plasmid were co-cultured with MDMs-Control or MDMs-lncMMPA in Transwell systems for 6 d and then collected for the indicated experiments. **F.** Lactate production of Hep3B cells with indicated treatments. **G.** Cell proliferation of Hep3B cells with indicated treatments.
